# Child and Adult Care Food Program Participation Benefits, Barriers and Facilitators for Independent Child Care Centers in California

**DOI:** 10.3390/nu14214449

**Published:** 2022-10-22

**Authors:** Danielle L. Lee, Elyse Homel Vitale, Samantha Kay-Daleiden Marshall, Christina Hecht, Lindsay T. Beck, Lorrene D. Ritchie

**Affiliations:** 1 Nutrition Policy Institute, University of California, Agriculture and Natural Resources, Oakland, CA 94607, USA; 2 CACFP Roundtable, San Diego, CA 92172, USA; 3 Nutrition & Food Services, University of California, San Francisco Medical Center, San Francisco, CA 94143, USA

**Keywords:** Child and Adult Care Food Program, child care, policy, benefits, barriers

## Abstract

The Child and Adult Care Food Program (CACFP) provides reimbursements for nutritious foods for children with low-income at participating child care sites in the United States. The CACFP is associated with improved child diet quality, health outcomes, and food security. However CACFP participation rates are declining. Independent child care centers make up a substantial portion of CACFP sites, yet little is known about their barriers to participation. Researcher-led focus groups and interviews were conducted in 2021–2022 with 16 CACFP-participating independent centers and 5 CACFP sponsors across California CACFP administrative regions to identify participation benefits, barriers, and facilitators. Transcripts were coded for themes using the grounded theory method. CACFP benefits include reimbursement for food, supporting communities with low incomes, and healthy food guidelines. Barriers include paperwork, administrative reviews, communication, inadequate reimbursement, staffing, nutrition standards, training needs, eligibility determination, technological challenges, and COVID-19-related staffing and supply-chain issues. Facilitators included improved communication, additional and improved training, nutrition standards and administrative review support, online forms, reduced and streamlined paperwork. Sponsored centers cited fewer barriers than un-sponsored centers, suggesting sponsors facilitate independent centers’ CACFP participation. CACFP participation barriers should be reduced to better support centers and improve nutrition and food security for families with low-income.

## 1. Introduction

Rates of food insecurity for families with children under six years old living in the United States in 2021 are higher compared to families with older children or no children [1]. Food insecurity also disproportionately impacts households with incomes below 185 percent of the poverty threshold, single-parent families, and Black and Hispanic families [1]. Food insecurity in childhood is associated with negative health outcomes and behavioral, academic, and emotional problems [2,3]. Given that most US children under six years old spend time in nonparental care, child care settings are an ideal environment to support health equity by improving nutrition and food security for young children [4,5].

The Child and Adult Care Food Program (CACFP) is the largest federal nutrition program that contributes to the healthy growth and development of young children in child care settings in the United States [6]. Since 1968, the CACFP provides free nutrition training, access to commodity foods, and reimbursements for nutritious meals and snacks to income-eligible children at participating child care sites. Meals served at CACFP-participating sites are often more nutritious and better aligned with recommended child feeding practices compared to non-CACFP participating sites [7,8,9,10]. CACFP participation has been shown to improve child diet quality, health outcomes, and food insecurity [11,12].

Despite the benefits of the CACFP, participation rates are declining and many eligible child care sites do not participate. Nationally, the CACFP provided over 435 million meals in family child care homes and 1533 million meals in child care centers in 2019. During the COVID-19 pandemic, these numbers dropped to 356 million and 1436 million, respectively in 2021 [13]. Although this steep decline is likely related to pandemic-related closures of child care sites [14], CACFP participation trends were steadily declining prior to the pandemic. Specifically in California, there was a decline in CACFP participation between 2010–2017: more than 1.7 million fewer meals were served in child care centers and 8 million fewer meals were served in family child care homes [15]. These are concerning trends given the critical role that the CACFP plays in improving nutrition and food security for children from families with low-income. Research to understand the barriers to participating in the CACFP is just beginning to emerge [16,17,18].

Administrative oversight of the CACFP at the state level is managed by state departments. In California, the Department of Social Services began providing administrative CACFP oversight during the global COVID-19 pandemic as of 1 July 2021, taking over this role from the California Department of Education [19]. To administer the CACFP, the state may contract directly with a child care entity or contract with private, non-profit, or community-based third-party intermediaries—called sponsoring organizations. Sponsors may be entirely responsible for the administration of the food program for (a) one or more family child care homes; (b) two or more centers; (c) a center that is a legally distinct entity from the sponsoring organization (this is a sponsor of independent centers) or (d) any combination of the aforementioned [20]. Sponsoring organizations may provide the entities they sponsor with software to help them submit their CACFP-related paperwork.

Independent centers are defined by CDSS as an agency that operates a center at a single physical site. They are independently owned and operated and not owned by a corporation. Independent centers can either work with a sponsoring organization to operate the CACFP at their site, or enter into an agreement with the CDSS to assume financial and administrative responsibility for the CACFP operations [21]. A nationwide survey of CACFP-participating centers suggests approximately a third of the centers in the US are independent with an average of 97 children enrolled per center [22].

Studies specifically focusing on independent centers’ experience with the CACFP are limited [18,22]. Interviews conducted of independent center stakeholders across the United States in 2018 identified health and nutrition for children, reimbursement, guidance and trainings as key benefits of participating in CACFP [18]. Key challenges included paperwork, time, and insufficient training and education [18]. In a 2017 national U.S. survey of CACFP-participating centers, independent centers were more likely to use smaller, local grocery stores to procure foods and beverages, likely resulting in unique barriers to CACFP-participation compared to centers procuring foods through a foodservice provider or warehouse store [22].

The aim of this study is to identify benefits, barriers, and facilitators in accessing the CACFP experienced by sponsors of independent centers and by independent centers themselves in California—both those that contract with CDSS to operate the CACFP and those that work with a sponsoring organization to operate the CACFP—as well as explore opportunities for improvement. Independent child care centers and sponsors were selected as the focus because relatively little is known about their experiences with the CACFP. Findings were elucidated through qualitative analysis of researcher-led focus groups and one-on-one interviews with independent centers and sponsors of independent centers. The goal of this study is to inform state CACFP agencies and the USDA on how to better support independent centers in accessing the CACFP.

## 2. Materials and Methods

### 2.1. Sample Selection, Recruitment, and Enrollment

In August 2021, CDSS provided researchers with a database of CACFP-participating agencies in California. From this database, a geographically diverse sample of three types were selected and recruited into separate focus groups: independent centers that contract directly with the state to operate the CACFP (FG1), independent centers that work with a sponsoring organization to operate the CACFP (FG2), and sponsors of independent centers (FG3). The goal was to recruit at least 10 participants per focus group, including at least one military or government organization and one serving tribal communities for groups (1) and (2).

To ensure diverse geographical representation, site contact zip-codes were matched to the USDA 2010 Rural-Urban Commuting Area (RUCA) codes. RUCA codes were then categorized into urban, suburban, and rural [23]. Child care centers were assigned a random number, sorted in ascending order, and then filtered by RUCA code. A quota sampling method based on the proportion of centers’ RUCA codes in the original CDSS database was used to select the final centers for recruitment.

Recruitment materials were sent via email by the CACFP Roundtable, a program contact, or a sponsoring organization. Interested participants were asked to contact researchers to be screened and enrolled in the focus groups. Inclusion criteria required that participants: (1) be the person who manages the CACFP, (2) be 18 years old or older, (3) have access to a computer, tablet, or smartphone, and (4) be at a site that participated in the CACFP as an independent center or a sponsor of independent centers during the last 5 years.

After initial focus groups were conducted, the sample was expanded to ensure representation from critical participants. NPI requested an additional CDSS CACFP database pull in January 2022 and used convenience sampling methods to identify and recruit 16 centers that had participated in the CACFP for 1 to 3 years. This was to ensure that FG1 was inclusive of centers recently enrolled in the CACFP. Additionally, 21 centers from the original FG2 sample were identified whose sponsors participated in FG3 and who had not yet received recruitment material or contacted researchers. Finally, one sponsor was identified for direct recruitment from the original sample. These centers and sponsors were contacted by researchers by telephone.

Ultimately, we screened 22 centers for FG1, 15 centers for FG2, and 5 sponsors for FG3 for enrollment in the study. All centers were eligible and agreed to participate. Many enrolled participants were unable to attend the scheduled focus groups or a one-on-one interview. Final participants included: (1) FG1—11 individuals from 10 centers; 2 individuals participated in one focus group, 4 in a second focus group, 4 in a third focus group, and 1 in an individual interview; (2) FG2—7 individuals from 6 center; 6 participated in one focus group and 1 in an individual interview; all centers were sponsored by organizations in FG3; (3) FG3—6 individuals from 5 sponsoring organizations; 4 participated in 1 focus group, and 1 in an individual interview; 2 sponsors had centers in FG2. We achieved our pre-determined goals for variation in participant characteristics. Table 1 summarizes sample sizes by focus group.

### 2.2. Surveys, Focus Groups and Structured Interviews

Enrolled participants—one from each center or sponsoring organization—were emailed a link to complete a 23-item (FG1/FG2) or 15-item (FG3) online survey (File S1) to capture demographics and site characteristics prior to participating in their scheduled focus group or interview. The surveys and interview guides were developed by researchers and reviewed by CDSS, the CACFP Roundtable, and CACFP-stakeholders. Topics included CACFP participation benefits, barriers, and what would help support CACFP participation. Questions were adjusted according to participants’ relationship with the CACFP (File S2). Participants received the questions (File S2) in advance of their scheduled focus group or interview, in addition to a glossary of terms commonly used in the CACFP. They were instructed to review these materials and seek answers to questions they were unable to answer themselves from other center or sponsor organization staff prior to attending their focus group or interview.

Each focus group session was led by a female peer-facilitator with over 20 years of experience as a center director working with the CACFP. A female PhD-level researcher (CH) from the research team also participated in the focus group sessions. The peer-facilitator was trained by CH on how to lead the focus groups. Each focus group lasted approximately 60–75 min and was conducted online using the Zoom video conferencing platform. Participants in all three groups (FG1, FG2 and FG3) were informed of the goal of the research project and the relationship of the interviewers to the project prior to answering questions. Participants who were unable to attend a scheduled focus group completed one-on-one interviews with the researcher using the same questions posed in the focus group. Focus groups and interviews were conducted December 2021 through March 2022. Participants received a $100 gift card for participating.

All discussions were audio-recorded, and field-notes were captured at the end of each focus group or interview. Each discussion was transcribed verbatim from audio recordings using Otter.AI (version 2022), then reviewed and cleaned by a researcher (DL) by removing vocal disfluencies–commonly known as filler words–and validating Otter.AI transcriptions against audio recordings. Transcripts were not returned to participants for comment or correction. Participant survey and focus group responses were given a unique ID to maintain confidentiality.

### 2.3. Data Analysis

Survey responses were analyzed using descriptive statistics in Microsoft^®^ Excel (version 16.62). The grounded theory method of analyzing qualitative data [24] informed transcript data coding, which was also conducted in Microsoft^®^ Excel (version 16.62). Two coders (LB, CH) developed an initial coding of themes by reviewing the transcripts to create unique codes that summarized the main points of each participant’s response. A separate coder (DL) reviewed the initial codes developed by LB and CH. Dissimilar codes were reconciled and incorporated into refined coding protocol. DL then reviewed and compared codes to determine connections and created subthemes. Key quotes were extracted to provide context for the selected codes. Codes were then summarized into thematic categories. This final step was reviewed by the other coders (CH, LB) and principal investigator (LR) for final revisions. Participants did not provide feedback on thematic results.

## 3. Results

### 3.1. Participant Characteristics

Participant and site characteristics are listed in Table 2. Participants were mostly the center owner, director or site supervisor, all were women, and most were white, Black or African American, Hispanic or Latinx. Most participants had a Bachelor’s degree or higher with English as their preferred language. Centers and sponsors were mostly non-profit organizations. All served preschool age children; some also served infant/toddler or school-age children. Centers were predominantly urban and participated in the CACFP from a range of 1 to less than 3 years up to 10 or more years. Centers and sponsors were located across all CDSS-established CACFP regions and most were in operation for 5 or more years. Two centers in FG1 had previously participated in the CACFP through a sponsoring organization, and one center in FG2 had previously contracted directly with the state to operate the CACFP.

Centers had an average of 14 staff. Sponsoring organizations had an average of 82 staff with 8 working in the CACFP departments. Most staff preferred using English. Children were predominantly English- or Spanish-preferring. A third to a half of the children at participating centers qualified for child care subsidies. Centers offered either full-day or both half- and full-day child care. The center director or supervisor was mostly responsible for both menu planning and CACFP administrative paperwork. One sponsored center worked with a sponsor that provided foodservice, and three FG3 sponsors provided their own foodservice. Nearly all centers offered at least breakfast and lunch, with several offering supper and some snacks throughout the day. Most centers prepared food on site at their center. Participants received support for the CACFP largely from the CACFP Roundtable (a non-profit organization), CDSS, or CDE (sponsors and centers that contract directly with the state). Centers working with sponsors mostly received support for the CACFP from their sponsoring organization.

### 3.2. Summary of Themes and Subthemes

**Figure 1:**
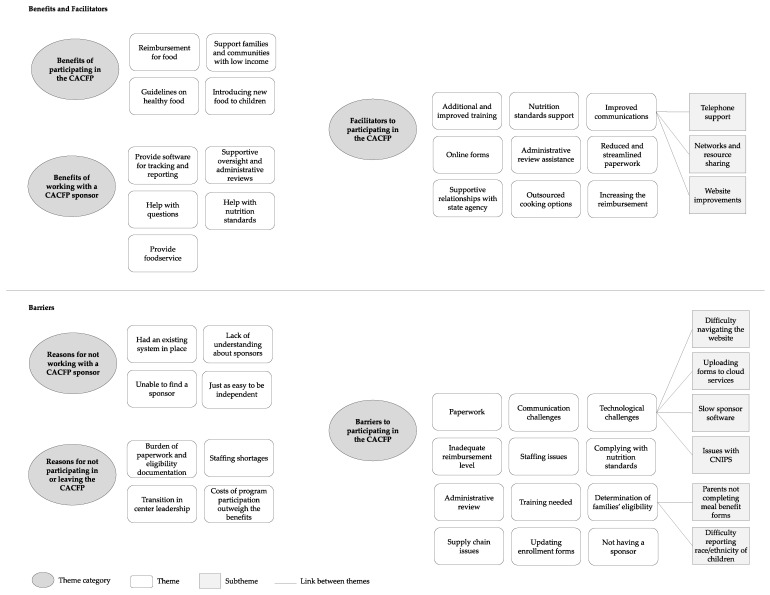
Emergent themes of benefits, barriers, and facilitators to participating in the Child and Adult Care Food Program (CACFP) and working with CACFP sponsors, as identified by independent child care centers and their sponsors located in California, United States.

Qualitative analysis resulted in six major thematic categories: (1) benefits of the CACFP, (2) benefits of working with a CACFP sponsoring organization, (3) barriers to participating in the CACFP, (4) reasons why some independent centers do not participate in the CACFP or have left the program, (5) reasons independent centers do not work with a CACFP sponsoring organization, and (6) facilitators to participating in the CACFP. Within each category there are overarching themes and several subthemes (Figure 1). 

#### 3.2.1. Benefits of Participating in the CACFP

Benefits of participating in the CACFP were numerous. Independent centers’ most cited CACFP benefit was reimbursement for food. Guidelines on healthy food and the ability to support families and communities with low-income also were often mentioned as benefits. For one center that works with a sponsoring organization, guidance was provided by a Registered Dietitian. Another center said that the CACFP facilitates them introducing new foods to children in their care. Themes are summarized with illustrative quotes in Table 3.

#### 3.2.2. Benefits of Working with a CACFP Sponsoring Organization

Independent centers that work with a sponsoring organization cited many benefits of that relationship. The most cited benefits included: sponsors provide software for tracking and reporting, support oversight and administrative reviews, help with questions, and help with nutrition standards. Additionally, one center reported that the largest benefit was that their sponsor provided foodservice—meaning the meals and snacks provided to children were prepared and delivered by the sponsoring organization. One center that contracts directly with the state to operate the CACFP (but who previously worked with a sponsoring organization) stated that one of their biggest barriers to participating in the CACFP was not having a sponsor. Themes are summarized with illustrative quotes in Table 4.

#### 3.2.3. Barriers Related to Participating in the CACFP

Independent centers working with a sponsoring organization generally cited fewer barriers compared to independent centers contracting directly with the state. Paperwork was the barrier most often cited by sponsors and centers that contract directly with the state. This barrier was not cited by sponsored centers. Communication challenges were the second most frequent barrier cited by sponsors and sponsored centers. This barrier was not cited by independent centers that contract directly with the state. Communication challenges related specifically to information that sponsors and sponsored centers received about the CACFP.

Technological challenges were commonly cited by both types of independent centers and by sponsors. These technological challenges often dovetailed with communication challenges. Challenges related to navigating the website, specifically finding forms, were common for centers contracting directly with the state. In California sponsors and centers contracting directly with the state use the Child Nutrition Information and Payment System (CNIPS) to process claims for reimbursement. While most independent centers contracting directly with the state reported having no issues with CNIPS, one independent center contracting with the state and all five sponsors reported CNIPS being difficult to use and having outdated information and infrastructure. Staff technology literacy was a challenge cited by one center contracting directly with the state.

Independent centers working with a sponsoring organization cited slow software provided by the sponsor as a technological challenge. However, one center found the sponsor-provided software more helpful than having to submit CACFP-related paperwork in written/paper form. Issues uploading forms to an online electronic file hosting and sharing service and the cost of this technology were challenges cited by both sponsoring organizations and sponsored centers.

Inadequate reimbursement and staffing issues were other frequently cited barriers reported by both sponsors and centers contracting directly with the state. These two barriers were cited as being interlinked as for centers the CACFP provides partial reimbursement for food costs and no reimbursement for staffing for meal preparation or administration of the program (however, administrative expenses are allowable for sponsors). These two barriers were not cited by sponsored centers. Within the theme of inadequate reimbursement, one sponsored center cited sponsor fees as a barrier to participating in the CACFP.

Several additional barriers were cited, though less frequently. Complying with nutrition standards was a barrier cited by independent centers and sponsors. Administrative review was a barrier cited by both types of centers. Eligibility determination was another barrier cited by centers. This was mostly related to difficulty getting parents to complete meal benefit forms due to their hesitancy to disclose their income or failure of parents to indicate their child’s race/ethnicity on the form. Two independent centers were curious why eligibility is dependent on family income and one center perceived the eligibility income threshold as too high. Updating enrollment forms due to changing child care numbers was a barrier cited by only one independent center contracting directly with the state (two centers contracting directly with the state said that child enrollment decreased during COVID-19). Training was a barrier cited only by centers contracting directly with the state. Supply chain issues were a barrier cited only by independent centers contracting directly with the state and sponsors of independent centers. These issues were largely due to COVID-19 pandemic-related supply chain inconsistencies. Themes and subthemes are summarized with illustrative quotes in Table 5.

#### 3.2.4. Reasons Why Some Independent Centers Do Not Participate in the CACFP or Have Left the Program

Independent centers and sponsors cited several reasons why some centers do not participate in the CACFP or have left the program. When asked if they had ever discontinued participation in the CACFP, a majority of centers said no. One center contracting directly with the state said they were considering leaving the program due to staffing shortages. Another said they had previously discontinued enrollment due to a transition in center leadership. Sponsors cited several reasons why independent centers do not participate in the CACFP or have left the program, such as the burden of paperwork and eligibility documentation, and the costs of CACFP participation outweighing the benefits. Themes are summarized with illustrative quotes in Table 6.

#### 3.2.5. Reasons Independent Centers Do Not Work with a CACFP Sponsoring Organization

Independent centers that contract directly with the state cited several reasons for not working with a sponsoring organization. Many did not operate the CACFP through a sponsor because they had an existing system in place that helped them manage the program. One said that it was just as easy to be independent. Other reasons were lack of understanding about sponsors or being unable to find a sponsor. Themes are summarized with illustrative quotes in Table 7.

#### 3.2.6. Facilitators to Participating in the CACFP

Improved communications was a major facilitator, containing several subthemes. Specifically, independent center participants requested telephone support and state website improvements, such as having a chat box on the state website. Sponsors also recommended having quarterly meetings among peers that allow for networking, shared resources, a dedicated CACFP website, and a more accurate and updated state CACFP listserv. Online forms were suggested by sponsors and both center types. Less paperwork and streamlined services were common suggestions by sponsors and sponsored centers. These themes were generally centered around tracking child enrollment and eligibility determination, and parents completing forms.

Centers commonly suggested continuing and/or expanding support for nutrition standards. Primary requests were for information on portion sizes, grocery shopping guides, sample menus, food recall information, and putting information in a digital format available online. Sponsors and centers mentioned improved training and administrative review assistance as additional facilitators. Two centers that contract directly with the state recommended having access to outsourced cooking options. A common theme for sponsors was the recommendation to have more supportive relationships with the state agency. Another recommendation to increase CACFP participation by independent centers was to increase the reimbursement rate. Themes and subthemes are summarized with illustrative quotes in Table 8.

## 4. Discussion

This study elucidated several benefits of, barriers to, and facilitators for participation in the CACFP by independent, licensed child care centers in California. A key benefit was offsetting the cost of nutritious foods served to children, particularly for low-income communities. However, many said that the amount of reimbursement was inadequate: it did not fully cover the cost of healthy meals or the necessary staff labor to support program administration. Despite appreciating the nutrition standards, some centers also found these to be a challenge: they didn’t fully understand the standards, they were unable to find foods that comply with these standards, children desired food that was not aligned with these standards, or they found it difficult to stay on top of changes to the standards. Both inadequate staffing and finding foods that adhered to the nutrition standards were heightened barriers during the COVID-19 pandemic.

Barriers related to the administrative work required to adhere to the CACFP were commonly cited by both centers and sponsors. Despite centers being able to submit paperwork online, the required paperwork was perceived as burdensome, particularly given levels of staffing. Specifically, eligibility determination and updating enrollment forms were perceived as barriers because families often were hesitant to disclose this information and centers did not want to guess race/ethnicity when not reported by parents. Communication and technological challenges dovetailed with the administrative barriers. These challenges stemmed from unclear communications from sponsors and the state agency about the CACFP requirements, difficulty navigating the state website to find the necessary forms, and difficulty uploading forms or providing information via CNIPS and online document sharing systems. Training staff on nutrition standards and administrative components was another barrier. Additionally, administrative reviews from the state agency that were perceived as punitive as opposed to supportive were cited as a barrier. Staffing shortages, burdensome paperwork, eligibility determinations, and the costs of participating in the CACFP outweighing the benefits were the main reasons why some centers opted to leave the CACFP, according to sponsors.

Although the COVID-19 pandemic is likely to have exacerbated barriers to participation, many of these same benefits and barriers have been cited by other child care centers outside of California prior to the pandemic. A 2019 survey of centers in Connecticut showed that centers of all types perceived the CACFP as an important program to support them in serving nutritious meals to children, particularly those without sufficient food at home [17]. This study also cited inadequate reimbursement and the high cost of nutritious food as barriers to all types of centers in participating in CACFP and to adhering to nutrition standards [17]. These centers also cited reporting requirements, burdensome paperwork and difficulty collecting income eligibility forms from families as barriers [17]. In a 2018 study of independent centers across the US, centers also cited reimbursement, providing nutritious meals to children and guidance on healthy food as benefits of CACFP participation [18]. They also similarly cited burdensome paperwork, inadequate staff time, training, communication issues, administrative reviews, adherence to nutrition standards, and availability of foods that align with nutrition standards as barriers to participation [18].

Independent centers with sponsors in our study cited fewer barriers to participating in CACFP. However, those that didn’t work with a sponsor said they didn’t know this was an option or were unable to find a sponsor. Given the important role sponsors seem to play in supporting the participation of independent centers in the CACFP, efforts should be taken to improve centers’ awareness of and access to sponsoring organizations.

Centers in our study cited several seemingly feasible opportunities to facilitate participation in the CACFP: improving state agency communications by offering telephone support, improving the state’s website, and offering sponsors networking and resource sharing opportunities. Additional and improved training, providing support on nutrition standards and administrative reviews, providing more supportive relationships to sponsoring organizations, providing information on how to access outsourced cooking options, reduced and streamlined paperwork, and providing online forms for parents were also recommended. Many of these recommendations were also common in CACFP-participating independent centers from across the US [18]. Some of these recommendations may be actionable as the congressional actions required to increase reimbursement may be less feasible for immediate implementation.

Efforts to improve nutrition in child care settings are not unique to the U.S., especially as a majority of young children in the 38 Organisation for Economic Co-operation and Development (OECD) member countries are in child care by the time they are 3 years old [25]. Two reviews of international, federal nutrition standards for child care in the United Kingdom, the U.S., New Zealand, and Australia suggest a lack of consistency in standards between countries [26,27]. Two studies evaluating adherence to national policy for nutrition in child care settings in England, where child care is free for all 3- and 4-year-olds, shows regional variations in the interpretation of federal policy and the implementation of voluntary federal nutrition guidelines for child care [28,29]. New Zealand government policies encourage nutrition guidelines in child care, but food served in child care is not subsidized like in the U.S. through CACFP [30]. Ireland provides voluntary food and nutrition guidelines for childcare, but little research has been done to determine adherence to these guidelines [27]. In Australia, no national guidelines for nutrition in child care exist, however jurisdictional food provision guidelines are in place but are inconsistent across jurisdictions [31]. Researcher in other countries have identified challenges similar to those identified in our California study in terms of difficulty meeting nutrition guidelines and difficulty in accessing training for implementing nutrition guidelines [28,30].

This study has several strengths. To our knowledge, it is the first study to look at the specific role sponsors of independent child care centers may play in alleviating barriers to CACFP participation. This study used a sampling method to ensure geographic and diverse representation of CACFP-participating independent centers and sponsors across the state. The study design of collecting qualitative data also provides more nuanced insight that survey research alone would be unable to accomplish.

This study has limitations. Given participants were only from California and a small subset of CACFP-participating sites, results may not be generalizable to CACFP-participating sites outside of California or sites other than independent child care centers. Despite attempts to recruit participants across all metropolitan regions, there were no sponsored centers from rural settings. Data were collected during the ongoing COVID-19 pandemic (December 2021–March 2022). As such, many potential participants may have been unable to participate given pandemic-related resource and time constraints, despite being offered a modest $100 incentive. Future studies should encourage participants to share barriers to CACFP participation that are not relevant to operations during a pandemic. Finally, although efforts were made to reduce social desirability bias, this is a general concern in qualitative research.

## 5. Conclusions

The CACFP supports improved nutrition and food security for young children with low-income by providing child care centers with reimbursement for healthy meals and snacks. Despite its perceived benefits by independent child care centers, several barriers to their participation in the CACFP exist. Compared to centers contracting directly with the state to operate the CACFP, sponsoring organizations appear to better support independent centers’ participation in the program. Numerous participant-recommended facilitators to CACFP participation exist. Efforts should be taken to implement these facilitators to reduce barriers to CACFP participation and increase independent child care centers’ awareness of and access to CACFP sponsoring organizations.

## Figures and Tables

**Table 1:** Child and Adult Care Food Program (CACFP) focus group recruitment sample and participation ^1^.

CACFP Group	Total in CDSS Database	Sent Recruitment Materials	Agreed to Participate	Participated
Independent centers contracting directly with the state (FG1)	342 (including 1 tribal and 5 military)	102 (including 1 tribal and 5 military)	22	10
Independent centers working with a sponsoring organization (FG2)	182 (0 tribal and 0 military)	100	15	6
Sponsors of independent centers (FG3)	10	10	5	5

^1^ CDSS—California Department of Social Services.

**Table 2:** California Child and Adult Care Food Program (CACFP) focus group participant and child care site or sponsoring organization characteristics ^1^.

Characteristic (*n* (%))	Focus Group 1 (*n* = 10)	Focus Group 2 (*n* = 6)	Focus Group 3 (*n* = 5)
**Job title**			
Center owner	4 (40)	4 (67)	--
Director or site supervisor	8 (80)	2 (33)	3 (60)
Other	1 (10)	1 (17)	4 (80)
**Sex, female**	10 (100)	6 (100)	4 (80)
**Race/ethnicity**			
American Indian or Alaskan Native	1 (10)	0 (0)	0 (0)
Asian or Other Pacific Islander	1 (10)	0 (0)	1 (20)
Black or African American	3 (30)	1 (17)	2 (40)
Hispanic or Latinx	2 (20)	1 (17)	1 (20)
White	3 (30)	4 (67)	1 (20)
**Education**			
High school graduate	0 (0)	0 (0)	1 (20)
Some college or Associates degree	4 (40)	1 (17)	1 (20)
Bachelor’s degree	3 (30)	5 (83)	0 (0)
Master’s degree or higher	3 (30)	0 (0)	3 (60)
**English preferred language**	10 (100)	5 (83)	5 (100)
**Site type**			
For profit	4 (40)	--	0 (0)
Government/Military	1 (10)	--	0 (0)
Non-profit	4 (40)	--	5 (100)
Tribal	1 (10)	--	--
**Program type**			
Infant/Toddler Center	6 (60)	2 (33)	--
Preschool Age Center	10 (100)	6 (100)	--
School Age Center	2 (20)	4 (67)	--
**Metropolitan areas served ^2^**			
Urban	8 (80)	5 (83)	--
Suburban	1 (10)	1 (17)	--
Rural	1 (10)	0 (0)	--
**CDSS CACFP Regions served ^3^**			
Northern	3 (30)	1 (17)	3 (60)
Central	3 (30)	2 (33)	1 (20)
Los Angeles	2 (20)	1 (17)	2 (40)
Southern	3 (30)	2 (33)	2 (40)
**Years in operation**			
1 to <3 years	1 (10)	1 (17)	0 (0)
3 to <5 years	1 (10)	1 (17)	0 (0)
5 to <10 years	1 (10)	0 (0)	3 (60)
10+ years	7 (70)	4 (67)	2 (40)
**Years of CACFP participation**			
1 to <3 years	3 (30)	3 (50)	--
3 to <5 years	0 (0)	2 (33)	--
5 to <10 years	2 (20)	1 (17)	--
10+ years	5 (50)	0 (0)	--
**No. staff in organization (Mean (SD))**	14 (7)	14 (8)	82 (100)
**Staff preferred language or Language organization supports for center directors (Mean % (SD))**			
English	80 (0.2)	79.5 (18.7)	5 (100)
Spanish	12 (0.2)	11 (9.6)	4 (80)
Chinese	3 (0.1)	2 (5.3)	0 (0)
Other	5 (0.1)	0 (0)	2 (40)
**No. children at center (Mean (SD))**			
0–5 months	1 (2)	1 (2)	--
6–11 months	3 (3)	1 (2)	--
12–23 months	8 (7)	7 (8)	--
24–35 months	11 (9)	21 (12)	--
3–5 years	34 (16)	51 (37)	--
6+ years	5 (10)	8 (12)	--
**Child preferred language (Mean % (SD))**			
English	70 (23.0)	79 (24.6)	--
Spanish	21 (25.0)	17 (20.4)	--
Chinese	5 (8.7)	4 (10.2)	--
Other	4 (6.3)	0 (0)	--
**Children qualify for subsidies (Mean % (SD))**	46 (29.0)	31 (15.4)	--
**Type of child care offered**			
Full day	4 (40)	1 (17)	--
Half- and Full day	6 (60)	5 (83)	--
**Responsible for menu planning**			
Director or site supervisor	6 (60)	5 (83)	--
Center teacher or teacher’s aide	0 (0)	0 (0)	--
Cook or chef	4 (40)	1 (17)	--
Dietitian	0 (0)	1 (17)	--
Other	1 (10)	1 (17)	--
**Responsible for CACFP administrative paperwork**			
Director or site supervisor	8 (80)	4 (67)	--
Center teacher or teacher’s aide	4 (40)	0 (0)	--
Cook or chef	3 (30)	0 (0)	--
Dietitian	0 (0)	0 (0)	--
Other	1 (10)	3 (50)	--
**Sponsor provides foodservice**	0 (0)	1 (17)	3 (60)
**Meals, snacks provided by center**			
Breakfast	8 (80)	6 (100)	--
Lunch	10 (10)	4 (67)	--
Supper	0 (0)	1 (17)	--
Mid-morning snack	6 (60)	0 (0)	--
Mid-afternoon snack	10 (100)	5 (83)	--
Evening snack	1 (10)	0 (0)	--
**Food preparation location (does not include food from parents)**			
On site (at center)	9 (90)	4 (67)	--
Central kitchen operated by center(s)	1 (10)	1 (17)	--
Other	0 (0)	1 (17)	--
**Response when asked about where they receive support for the CACFP**			
CACFP Roundtable	4 (40)	0 (0)	4 (80)
National CACFP Sponsors Association	0 (0)	0 (0)	1 (20)
National CACFP Forum	1 (10)	0 (0)	2 (40)
USDA Team Nutrition	1 (10)	0 (0)	3 (60)
Institute of Child Nutrition	0 (0)	0 (0)	1 (20)
CDSS or CDE	5 (50)	0 (0)	5 (100)
Other	0 (0)	1 (17)	0 (0)

^1^ Focus group 1 included independent child care centers that contract directly with the state to operate the CACFP. Focus group 2 included independent child care centers that work with a CACFP sponsoring organization. Focus group 3 included sponsors of independent child care centers that operate the CACFP. The double dash (--) indicates not applicable because it does not apply or because these questions were not asked. CDSS—California Department of Social Services. CDE—California Department of Education. USDA—United States Department of Agriculture. ^2^ Categorized by assigning the USDA 2010 Rural-Urban Commuting Area (RUCA) codes matched to the center zip code. RUCA codes were then categorized into urban, suburban, and rural classification. ^3^ Categorized based on the self-reported counties served. CDSS CACFP administrative regions include: Northern, Central, Los Angeles, and Southern. Northern region California counties include: Alpine, Amador, Butte, Calaveras, Colusa, Del Norte, El Dorado, Glenn, Humboldt, Lake, Lassen, Marin, Mendocino, Modoc, Mono, Monterey, Napa, Nevada, Placer, Plumas, Sacramento, San Benito, San Joaquin, San Luis Obispo, Santa Clara, Santa Cruz, Shasta, Sierra, Siskiyou, Sonoma, Sutter, Tehama, Trinity, Yolo, Yuba. Central region California counties include: Alameda, Contra Costa, Fresno, Inyo, Kern, Kings, Madera, Mariposa, Merced, San Bernadino*, San Francisco, San Mateo, Solano, Stanislaus, Tulare, Tuolumne. Los Angeles region California counties include: Los Angeles*, Santa Barbara, Ventura. Southern region California counties include: Imperial, Los Angeles*, Orange, Riverside, San Bernardino*, San Diego. * Indicates counties located in two regions.

**Table 3:** Emergent themes of benefits of participating in the Child and Adult Care Food Program (CACFP) ^1^.

Theme	Illustrative Quotes
Reimbursement for food	*“Probably the main reason why we participate in the program [CACFP] is to subsidize the cost.”*—FG1_6
	*“I wanted to make sure to be able to continue to provide lunches for the children and not have to increase the parents’ tuition to cover the cost of that.”*—FG1_9
	*“It [CACFP] helps us offset the cost of the food, the cost of food is rising…”*—FG2_1
Supporting families and communities with low income	*“The kids that typically attend here are low-income and on subsidy programs… it really helped the families.”*—FG1_8
	*“[We have] a large number of students on low income, subsidized, who needed the lunch program.”*—FG1_11
Guidelines on healthy food	*“We like the guidelines for food. It keeps healthy food in our center, as opposed to having parents bring whatever they want, which may not be nutritious.”*—FG1_2
	*“I like the technical assistance. I like knowing that I’m serving the correct portions, the enhanced menu items, the different kinds of vegetables and whole grains.”*—FG1_1
	*“They [the sponsor] have a dietitian that helps look over our menus just to validate the nutrition level and monitor all the food groups… and keeping us on track with the healthiest options that we can serve.”*—FG2_3
Introducing new foods to children	*“…introducing new foods to kids, which is great, because I have picky eaters.”—*FG2_3

^1^ Identified by focus group participants which included independent child care centers contracting directly with the state to operate the CACFP (FG1, *n* = 10), independent child care centers that operate the CACFP through a sponsoring organization (FG2, *n* = 6), and sponsors of independent child care centers that participate in the CACFP (FG3, *n* = 5). Letters/numbers after each quote represent the source of quote (e.g., FG1_1 was participant assigned the participant ID as number 1 in the focus group of independent child care centers contracting directly with the state).

**Table 4:** Emergent themes of benefits of working with a CACFP sponsoring organization ^1.^.

Theme	Illustrative Quotes
Provide software for tracking and reporting	*“[The sponsor] provides us with a system called Kid Kare that helps to keep track of all of the food that we’ve served.”*—FG2_2
Supportive oversight and administrative reviews	*“They [the sponsor] see if there was anything that we might have missed, like expired enrollment forms are an easy one to miss.”*—FG2_2
	*“They [the sponsor] also oversee the audits [administrative reviews]. They come in and kind of do little control checks on our service, cleanliness, temperature checks, lots of things. They help us ensure that we have the accurate meal benefit forms filled out and that we’re accessing the reimbursements for all the kids. And they help process the payments.”—*FG2_3
Help with questions	*“They’re [the sponsor] always there if I need to reach out and have questions.”*—FG2_4
Help with nutrition standards	*“They [the sponsor] also help with providing us with the food service guidelines, and they give us lots of handouts and just healthy meal ideas.”*—FG2_2
Provide foodservice	*“We receive our food from a food vendor [the sponsor] … we do the count, and we give them the count. They have the children’s names, and they bring the food and serve breakfast, lunch and snacks every day.”—*FG2_7

^1^ Identified by focus group participants which included independent child care centers contracting directly with the state to operate the CACFP (FG1, *n* = 10), independent child care centers that operate the CACFP through a sponsoring organization (FG2, *n* = 6), and sponsors of independent child care centers that participate in the CACFP (FG3, *n* = 5). Letters/numbers after each quote represent the source of quote (e.g., FG1_1 was participant assigned the participant ID as number 1 in the focus group of independent child care centers contracting directly with the state).

**Table 5:** Emergent themes of barriers to participating in the CACFP ^1^.

Theme	Illustrative Quotes
Paperwork	*“My challenge is the paperwork… getting it uploaded to the site… for me, it’s just not very intuitive.”*—FG1_6
	*“We implement a lot of technological resources to reduce paperwork. But for those centers that are still running, with paper driven operations, it’s paperwork. There’s a lot of moving pieces, a lot of ways to mess up, and small technological, for small technical aspects of the program that can end up costing the center their reimbursements.”—*FG3_5
	*“I have no program [for streamlining the paperwork]. I have to do everything, write it out… I’ve been doing the claims and everything by hand.”*—FG3_6
Communication challenges	*“So sometimes going back and forth over email, they’ll [the sponsor] send me an email, sometimes, I may not understand what they’re talking about. So, it’s a lot of back-and-forth emails.”—*FG2_1
	*“I would say the greatest technological barrier is information, transferring of information, receiving information, receiving two different answers for the same question from two different people [State analysts].”*—FG3_2
	*“I’ve been starting to get these emails where they [state agency] start sending you these things like notices, and I’ll click into the links, but I still have trouble where to be guided on what to click on to read what they are telling me… I go to the link, and I still am unable to locate where that information is.”*—FG3_6
Technological challenges	
Subtheme 1. Difficulty navigating the website	*“…when I go to find a form. They’re not alphabetical. They’re listed by form number. And it’s so frustrating to have to read all of those to find the one I want because it wastes so much time.”*—FG1_1
	*“I would like to be able to access any new policy or new procedures that we have more easily. I mean, I’ve searched for like an hour or so. And I didn’t find anything today.”*—FG1_5
Subtheme 2. CNIPS difficult to use, has outdated information and infrastructure	*“I rarely go to that website [CNIPS]. Because it’s hard. For me, the whole program is kind of difficult, because they send out things and they have you do these classes once a year, but they’re not really helpful.”*—FG1_3
	*“[I wish] that you could just keep the previous year’s information [in CNIPS] and not have to go back… a lot of the stuff is a repeat.”—*FG3_3
	*“I think there’s ways that for a sponsor, it [CNIPS] can be more streamlined. There’s a lot of bottlenecks between submission to the state agency, and then the timeframe it takes for an analyst to get back to the sponsor… for timing purposes, it becomes a little difficult.”—*FG3_5
Subtheme 4. Slow software provided by sponsoring organization	*“The system [Kid Kare software] is very, very slow. And so, it’s very time consuming.”—*FG2_4
	*“It’s very slow…but there’s a lot of [data] input that you have to put in for each child and it can take a long time to do that when the program [Kid Kare by Minute Menu] is running very slow.”—*FG2_5
Subtheme 5. Uploading forms to cloud-based file hosting service ^2^	*“And we’ve had to create Dropboxes now. There’s just a little bit more expense when you add the technological piece. So, sometimes building into your budget, I had to add more staff, if we were going to add the technology piece, and then the equipment piece and the cost. I mean, five Dropboxes, and then you add 40 Dropboxes, it does add a little bit.”*—FG3_1
Inadequate reimbursement level ^3^	*“We came about $4000 shy of being fully reimbursed for the food we served, much less any of the staff salaries or equipment or expenses.”—*FG1_1
	*“It’s sufficient [the reimbursement], but it doesn’t cover everything. It doesn’t cover the time for the work being done. Paperwork or the cook.”—*FG1_2
	*“I think it [the reimbursement level] should be a little higher, because food is very expensive… they’re asking you to buy these products, certain products, but it’s really hard to be able to have enough money to buy the better products…because it’s expensive.”—*FG1_3
	*“I wish that we would be reimbursed for everybody. It would be nice because it’s overall beneficial for all the kids and just because a child’s income level is higher it doesn’t mean they’re eating a balanced meal at home at all or exposed to different kinds of foods.”—*FG2_5
Staffing issues	*“We were short-staffed… we didn’t have enough staff and found that the time that goes in to do the administrative work and counting correctly and training [is a barrier].”*—FG1_10
	*“The staffing has been just a nightmare [during the COVID-19 pandemic] … we’re just not able to find staffing… my directors have literally ended up doing cooking and cleaning to maintain this program.”—*FG1_11
	*“The staffing… [is a barrier centers experience when participating in CACFP].”*—FG3_2
Complying with nutrition standards	*“So, I’ve looked at their [the CACFP] buying guides, their meal patterns… I still find them both to be very difficult when I go to the store to choose what they’re saying that I can do, or just finding foods. Especially now finding foods that fits into the categories that they want.”—*FG1_3
	*“One of the biggest challenges is the constant changing of what we can serve or what we can’t serve.”—*FG2_2
	*“Some of the things that the kids like, is not something that they’re able to prepare for the kids just because of the rules that they [the CACFP] have to follow… sometimes it doesn’t give you all the flexibility that you like.”—*FG2_7
	*“The ability to have the centers that we serve understand the processes. We have such a hard time just getting across simple requirements, that CDE and now CDSS pass on to us that we must pass on to them. And it’s a barrier because even though they want the food. We can’t get the buy in from the centers.”—*FG3_2
Administrative review	*“The very first review that I had after my first year was extremely intimidating, because I didn’t ever have anyone come in because of the pandemic and show me anything. So, we were trying to do this* via *email and whatnot.”—*FG1_9
	*“It’s kind of hard on the monitoring [administrative review] … [the sponsor] only have a sampling of some of us who will be chosen for the monitoring [administrative review]. So, we don’t actually always see the state [agency]. But that’s why they come out and do their checks, and kind of do their own version of it. So, I only see it speaking for myself through the eyes of the sponsor”—*FG2_3
Determination of families’ CACFP eligibility ^4^	
Subtheme 1. Difficulty reporting race/ethnicity of children	*“One of the challenges that came up on my last review was having to put a number for the nationality/race information. On the packet that you give to the parents, it states that portion is optional to fill out…. Upon review, I told her [CACFP state monitor] I don’t have an exact number, because we have families that I don’t feel comfortable assuming I know what they are. We have many Native Americans and honestly looking at them, I wouldn’t be able to assume that. So, I was really uncomfortable giving that information to them [CACFP state monitor] … I said, “Some of them opted out, they didn’t fill in the information.” And she basically told me to guess, and I don’t think that’s fair to put on us.”—*FG1_9
	*“I was told that we weren’t we were not supposed to discriminate, but then they’re [CACFP state monitor] making us guess [race/ethnicity]. But I can’t get at somebody’s nationality or ethnicity based on what they look like. And that’s discrimination in itself I thought. Either they should just not ask us, not force us to guess on it… or just not ask at all.”—*FG1_10
Subtheme 2. Parents not completing meal benefit forms	*“A lot of parents don’t want to complete the form [meal benefit form] because they don’t want to put their income down.”—*FG2_4
Training needed	*“Understanding it to the level that we feel that we’re confident and can do it right. So, for me, that has been very hard to make sure that my cook knows what to do, my teachers know what to do, as well as myself, the administration part. Because of the pandemic, we’ve had very little contact with the state [agency] at all as to what to do. And this is our first time, so it’s very tough.”—*FG1_8
	*“Training is always an issue. Our teachers have the basic training, and they don’t think [nutrition] is as important as everything else.”—*FG1_2
Supply chain issues	*“We can’t find certain items at the grocery store… we want to buy the whole grain pasta. They don’t have it, or there’s just certain things sometimes the store runs out of. So, we go to grocery stores, we don’t have Sysco deliver or anything like that… And sometimes they don’t have the items on the shelves. So that is a challenge… Also, even paper products like plates and cups and spoons run out, too, not just food, but items that we need for the food program.”—*FG1_4
	*“It’s been hard for certain vendors to get certain products, so it’s been a little dicey with certain items to use lately.”—*FG3_6
Updating enrollment forms	*“It’s just keeping up with all of the dates, when children withdraw. We get a lot of withdrawals and re-enroll.”—*FG1_8
Not having a sponsor	*“One of my greatest challenges is being my own sponsor. It’s because I’ve also done it the other way, I know how they both work, it is a lot more work [not participating in the CACFP through a sponsor].”—*FG1_9

^1^ Identified by focus group participants which included independent child care centers contracting directly with the state to operate the CACFP (FG1, *n* = 10), independent child care centers that operate the CACFP through a sponsoring organization (FG2, *n* = 6), and sponsors of independent child care centers that participate in the CACFP (FG3, *n* = 5). Letters/numbers after each quote represent the source of quote (e.g., FG1_1 was participant assigned the participant ID as number 1 in the focus group of independent child care centers contracting directly with the state). CNIPS—Child Nutrition Information and Payment System—the online database used by the CACFP administrative state agency, California Department of Social Services (CDSS) to receive and maintain agency applications and to process claims for reimbursement. ^2^ During the COVID-19 pandemic, CACFP-participating agencies were temporarily asked to submit annual review forms (e.g., meal count forms, intake forms, enrollment forms) online by uploading them to a cloud-based file hosting service (Dropbox) managed by CDSS. ^3^ Child care centers are reimbursed different amounts for food depending on whether children quality for free, reduced-price or fully paid. ^4^ Reporting child race/ethnicity is a CACFP requirement from the USDA.

**Table 6:** Emergent themes of reasons why some independent centers do not participate in the CACFP or have left the program ^1^.

Theme	Illustrative Quotes
Burden of paperwork and eligibility documentation	*“It’s the paperwork… Just having a center allow us to look at their enrollment records is a task… Because once we start asking for paperwork… for meal benefit forms to be completed, they [centers] want to opt out, or [centers say] “I don’t want the service… we can’t afford to be a center that won’t comply”. But then that’s why they [centers] pull back because we request too much information from them in their eyes. Or too much at one time, I guess I should say… or the parents don’t want to complete the forms [meal benefit form, enrollment form]. They [parents] don’t want you to have their information.”—*FG3_2
	*“And then also eligibility documentation, a lot of parents don’t understand it, they don’t want to fill it out. And they’re less than forthcoming about the information on the eligibility documents… “–* FG3_5
Staffing shortages	*“We’re serving about 90 lunches a day. And when my directors are being pulled in to do that, the rest of the school is falling apart. So, we considered very seriously in this last month, not continuing with [the] program [CACFP] just because we don’t have the manpower to keep administering it… all of the manpower and payroll involved is starting to become just not just worth it, not doable, not sustainable. So, we’ve reconsidered the food program recently.”—*FG1_11
Transition in center leadership	*“When I came, they had discontinued [participating in the CACFP] …I think it’s because they did a transfer of staff, of directors…So, I decided to start it back up.”—*FG1_5
Costs of program participation outweigh the benefits	*“If they’re [centers] not staying abreast of what they’re doing wrong, a review of three months of paperwork can turn into 12 months and go back three years. And so, all of a sudden, now they [centers] owe money back to the state agency, and they’re looking at it like, ‘Well, this was a lot of work, really, for nothing in the end, and it cost me money. So why continue to go forward with it?’”—*FG3_5

^1^ Identified by focus group participants which included independent child care centers contracting directly with the state to operate the CACFP (FG1, *n* = 10), independent child care centers that operate the CACFP through a sponsoring organization (FG2, *n* = 6), and sponsors of independent child care centers that participate in the CACFP (FG3, *n* = 5). Letters/numbers after each quote represent the source of quote (e.g., FG1_1 was participant assigned the participant ID as number 1 in the focus group of independent child care centers contracting directly with the state).

**Table 7:** Emergent themes of reasons independent centers do not work with a CACFP sponsoring organization ^1^.

Theme	Illustrative Quotes
Had an existing system in place	*“The school used to be a Head Start state preschool, and they’ve dealt directly with the state so we just kind of followed in line with the previous blueprint of the school.”—*FG1_8
	*“We just took over from a school and adopted what they were doing, they did all their own administration of the program, they kind of trained us on how to do it.”—*FG1_11
Lack of understanding about sponsors	*“In [my county], only family child care providers could use the sponsoring agency.”—*FG1_1
	*“I wasn’t aware of the other sponsoring agencies out there… had I known that there was a sponsor, I probably would have preferred that over dealing directly with the state.”—*FG1_8
Unable to find a sponsor	*“I was told that I had to be my own sponsor. That was the only option because I was not family child care any longer.”—*FG1_9
	*“I talked to two different sponsors. A lot of them are just serving family centers (family child care homes), not center based. So, I wasn’t able to find anybody who could help us. I just decided to learn it myself.”—*FG1_10
Just as easy to be independent	*“[Our director] did have a sponsoring agency. And then she figured out that she was still doing all the work and providing data to them. So, then she just figured that she could just do it herself. So, she became independent.”—*FG1_4

^1^ Identified by focus group participants which included independent child care centers contracting directly with the state to operate the CACFP (FG1, *n* = 10), independent child care centers that operate the CACFP through a sponsoring organization (FG2, *n* = 6), and sponsors of independent child care centers that participate in the CACFP (FG3, *n* = 5). Letters/numbers after each quote represent the source of quote (e.g., FG1_1 was participant assigned the participant ID as number 1 in the focus group of independent child care centers contracting directly with the state).

**Table 8:** Emergent themes of facilitators to participating in the CACFP ^1^.

Theme	Illustrative Quotes
Improved communications	
Subtheme 1. Telephone support	*“One time I was calling for something… I thought it was our analyst number, and it said, ‘I’m not available if you need help call this person, this person’… I call those numbers and it said, ‘If this is an emergency and you need an answer right away, call this person.’ It kept giving me phone numbers. And I was like, ‘Wow!’”—*FG1_4
	*“If there’s an open helpline where somebody could just answer a phone call to answer a simple question that we have, when we’re either filling out a paper for paperwork or whatnot, on the regulations and procedures, I think that would really help.”—*FG1_9
	*“I think for us, a lot of things have to do with you can’t call them and talk to them. Everything is* via *email… it’s a lot of back-and-forth emails, and then there’s really no support over the phone.”—*FG2_1
Subtheme 2. Networks and resource sharing	*“Maybe have quarterly meetings with all of us. Because we’re all experiencing many similar things…So with the CACFP, that would be very helpful [for them to] regionalize. Even if we were all to get on a Zoom meeting together and [say], “You are region one, you are region two, you are region three,” and we all would have one liaison or analysts that we can go to for technical and training assistance… There are so many divisions and arms under CDSS. So, if the CDSS CACFP had its own website… and update the listserv.”—*FG3_1
	*“I was fortunate enough to be on a coalition…it pulled a whole bunch of us together to say, ‘Who are we missing? How can we reach the people that aren’t participating? And can we develop them into their own sponsors?’… I would love to see that for California, to know who else is out there. Or maybe we do Northern California and Southern California, we can split it up. But I think that’s great, because if nothing else, know that you’re not alone… So, anything where you can interact with the state and other organizations that are involved”—*FG3_4
Subtheme 3. Website improvements	*“The website could be better. It’s just not as user friendly as I’d like.”—*FG1_2
	*“I think a chat option [on the website] would be kind of nice.”—*FG1_4
Additional and improved training	*“I do appreciate that they have a lot of stuff on YouTube for the CACFP and different little things that I have watched…. but having some in-person training. There’s nothing like it, then having somebody to actually converse with.”—*FG1_8
	*“Because my director had she not had experience at our previous school, I probably would have not kept moving forward with it [the CACFP]. Because it would seem so daunting. And there was just nobody to really… nobody could explain it to me. I feel like some of the trainings I took were not helpful in the administration of the program. It was like, ‘Great, I’m learning about portions and nutrition.’ But it didn’t really break down a lot of the administrative stuff that I had to do.”—*FG1_11
	*“If there were common questions that a lot of people have that maybe they can do like a video on a step-by-step or go over that and then we’re not having to read a huge book.”—*FG2_5
	*“In the beginning [when initially participating in the CACFP] more hands-on training…”—*FG1_9`
	*“If the state [CDSS] could maybe develop some kind of a video or webinar, a walk-through orientation…”—*FG3_1
	*“It’s hard to effectively run the programs, when the changes that are being made, there’s no training.”—*FG3_2
Nutrition standards support	*“The recipes, all the emails, I read them all… We get a lot of information to be able to continue to be effective, and in a meal planning, and keeping the kids having different recipes, healthy ways to prepare the food. Everything you guys send is very, very helpful…everything’s online, the Food Buying Guide, all those things that are online, those resources are really very good. Because they’re on our computers. It’s not a big book anymore that we have to handle. Those resources are great.”—*FG1_4
	*“Their [the state agency’s] explanation…is difficult to translate from what they’re saying to what you’re actually reading in the grocery store… examples of menus. I feel like I have no understanding when I go into their website of how to make sure that they’re getting the right amount…”—*FG1_3
	*“It would be extremely helpful to get a sample menu or a couple of different meal suggestions on not only what they’re [the USDA] changing, but also a balance of the other components of a meal in terms of what they see as being a really healthy balance. And even some menu suggestions.”—*FG2_3
Online forms	*“I wish [the enrollment forms] could be more electronic. …being able to have [the things that we have to submit monthly] on a digital platform and we can fill that information out and just as many forms as possible over into the digital world than paper would be great.”—*FG1_8
	*“The [form] that lists the children’s names and their eligibility like free reduced, what have you, that would be great if we could, that can be like saved in the program somewhere… As of right now I go through, I type in everyone’s name. And then the next month, if I add anyone, I have to type a whole new one. It would be nice if there was like an electronic way to have that in there that made it simpler to add and delete children, and then print that out for each month.”—*FG1_9
	*“If there was some type of app that [parents] could just fill out from their phone with the paperwork that might be easier.”—*FG2_7
Administrative review assistance	*“I think the best technical assistance we get is during the audits [administrative reviews] … They also provide us with really important information that every auditor [reviewer] has given different perspectives on different things and they’ve all enhanced how we were able to perform for the next audit [review].”—*FG1_1
	*“…when I first got audited [reviewed], how to better prepare somebody for that.”—B6*
	*“I think it would be very helpful that we get the instrument that says, ‘Here’s what the Administrative Review [is], here’s all the documents, here’s your evidence, and so on and so forth.’”—*FG3_1
	*“…training before the Administrative Review.”—*FG3_1
Reduced and streamlined paperwork	*“…be able to continue streamlining services so that it’s not new methods. There’s so much that always changes and that were responsible for. So, trying to keep it streamlined… just making it a little more user friendly…. making it quicker and a little easier for us, because we have a lot on our plate.”—*FG2_3
	*“…with our CACFP, the child care enrollment… all the things that we have to look at, at the center, in order to sponsor them. Because they’re now under CDSS. If that information is approved for CDSS, we shouldn’t have to go back and double check and triple check… CDE would ask us to verify enrollment for each child [for CDSS child care licensing]. It seems like we have to do now two extra steps for the daycare center just to be sure that they qualify for the CACFP when they already qualified for the CACFP. But then we have to now do an extra step to go through their records, to see that we have everything we need. And I think if they’re already under CDSS, I don’t see why we should have to do the extra step.”—*FG3_2
	*“I could go on and on about streamlining services, getting rid of half the paperwork that exists.”—*FG3_4
Supportive relationships with state agency	*“…having some field visits, not so much of a compliance, regulatory, “I got you”, but rather, “Wow, this is a great job, this is right, you’re on the right track.” And then providing links directly and resources for those centers.”—*FG3_1
	*“It would be very helpful if information came to us not in the way of “Here’s what you did wrong.” But to prevent us from doing something the wrong way, or in a non-compliant way, because it’s not intentional.”—*FG3_2
	*“I really had a good relationship with several of them [state agency analysts], and I felt like I could call and get a good answer, and I could get the support I needed… it’s the unknown right now of what it’s going to look like as we move forward… it’s just building up those relationships again… instead of getting my hand slapped. If it was just, ‘I just want to come and see you and see how you’re doing and see how I can help you.’ Rather than the ‘No, no, no.’… I think there’s also the whole image, like a marketing image. So, people aren’t so frightened to participate [in the CACFP], that they will get their hand slapped. I think that more partnership with the Food Program, the USDA and the state of California wants us to feed children, and we all want children to be healthy, and developmentally capable, and all these other things, so that they can learn and grow and be healthy, and productive members of society.”—*FG3_4
	*“…leading with a softer approach and understanding. Not necessarily coming in and wielding a sword is the best option. The state agency probably needs to support the sponsors more because we have the ability to be more involved [with centers] than the state agency can.”—*FG3_5
Outsourced cooking options	*“I know that some places do the food delivery through a centralized kitchen…I would love to do that…would be so much easier to just outsource it. And I don’t know how to do that.”—*FG1_1
Increasing the reimbursement	*“Give more money toward it!”—*FG1_6
	*“More money coming towards, what we get back, what we get.”—*FG1_7

^1^ Identified by focus group participants which included independent child care centers contracting directly with the state to operate the CACFP (FG1, *n* = 10), independent child care centers that operate the CACFP through a sponsoring organization (FG2, *n* = 6), and sponsors of independent child care centers that participate in the CACFP (FG3, *n* = 5). Letters/numbers after each quote represent the source of quote (e.g., FG1_1 was participant assigned the participant ID as number 1 in the focus group of independent child care centers contracting directly with the state).

## Data Availability

The data presented in this study are available within the article in Table 2 and Table 4.

## Supplementary Materials

The following supporting information can be downloaded at: https://www.mdpi.com/article/10.3390/nu14214449/s1, File S1. Survey questions. File S2. Focus group/interview questions.
