# Supplementary material for: Child and Adult Care Food Program Participation Benefits, Barriers and Facilitators for Independent Child Care Centers in California
Source: Nutrients. 2022 Oct 22;14(21):4449. doi: 10.3390/nu14214449 (PMC9654096; doi:10.3390/nu14214449)
Supplement: Supplementary file 1 [file nutrients-14-04449-s001.zip › File S1.pdf]

Survey Questions

A.1. Survey for Focus Group 1 – Independent Child Care Centers that Contract Directly with the State to Operate the CACFP and Focus Group 2 – Independent Child Care Centers that Operate the CACFP Through a Sponsoring Organization

1. Thank you for agreeing to participate in the California Department of Social Services Child and Adult Care Food Program transition focus group. As a reminder, we provided you a letter of information over email detailing the goals of this research study. Here is a link to that letter.
   Please complete this brief online survey before you participate in the online Zoom video conference focus group.
   Please click the arrow, below, to begin the survey.

2. Please answer the following questions about your child care site.

3. What is the zip code of your organization?

4. Which counties does your organization serve? (choose all that apply)
   Alameda (1)
   Alpine (2)
   Amador (3)
   Butte (4)
   Calaveras (5)
   Colusa (6)
   Contra Costa (7)
   Del Norte (8)
   El Dorado (9)
   Fresno (10)
   Glenn (11)
   Humboldt (12)
   Imperial (13)
   Inyo (14)
   Kern (15)
   Kings (16)
   Lake (17)
   Lassen (18)
   Los Angeles (19)
   Madera (20)
   Marin (21)
   Mariposa (22)
   Mendocino (23)
   Merced (24)
   Modoc (25)
   Mono (26)
   Monterey (27)
   Napa (28)
   Nevada (29)
   Orange (30)
   Placer (31)
   Plumas (32)

Riverside (33)
Sacramento (34)
San Benito (35)
San Bernardino (36)
San Diego (37)
San Francisco (38)
San Joaquin (39)
San Luis Obispo (40)
San Mateo (41)
Santa Barbara (42)
Santa Clara (43)
Santa Cruz (44)
Shasta (45)
Sierra (46)
Siskiyou (47)
Solano (48)
Sonoma (49)
Stanislaus (50)
Sutter (51)
Tehama (52)
Trinity (53)
Tulare (54)
Tuolumne (55)
Ventura (56)
Yolo (57)
Yuba (58)

5. What is the total number of staff at your organization (counting yourself?)

6. How many of your staff (including yourself) prefer the following for their primary language? Please be sure that the total % of all languages adds up to no more 100%.
English (1)
Spanish (2)
Chinese (3)
Other (4)

0% (none (0)
Less than 25% (1)
25-49% (2)
50-74% (3)
75-99% (5)

7. What is the total number of children at your center?

8. What is the total number of children at your center by age? (enter a '0' if none)
0-5 months (1)
6-11 months (2)
12-23 months (3)
24-35 months (4)
3-5 years (5)

6 years and older (6)

9. How many children cared for at your center prefer the following for their primary language? (enter a '0' if none). Please be sure that the total % of all languages adds up to no more 100%.
   English (1)
   Spanish (2)
   Chinese (3)
   Other (4)

   0% (none (0)
   Less than 25% (1)
   25-49% (2)
   50-74% (3)
   75-99% (5)

10. How many children currently cared for at your center qualify for child care subsidies?
    None (0)
    <25% (1)
    25-49% (2)
    50-74% (3)
    75-99% (4)
    100% (5)
    Don't know (6)

11. What type of child care does your site offer?
    Full-day (1)
    Half-day (2)
    Both full- and half-day (3)

12. Who is responsible for menu planning? (choose all that apply)
    Center teacher or teacher's aide (1)
    Director or Site Supervisor (2)
    Cook or chef (3)
    Dietitian (4)
    Other (write in) (5)

13. Who is responsible for CACFP administrative paperwork? (choose all that apply)
    Center teacher or teacher's aide (1)
    Director or Site supervisor (2)
    Cook or chef (3)
    Dietitian (4)
    Other (write in) (5)

14. Which meals and snacks are provided? (choose one answer per line)
    Breakfast (breakfast)
    Lunch (lunch)
    Supper (supper)
    Mid-morning snack (snack_m)
    Mid-afternoon snack (snack_a)
    Evening snack (snack_e)

Not provided (0)
Usually provided by center (1)
Usually brought from home by parents (2)

15. How is food prepared at your center? (do not include food brought in by parents)
    Prepared on site (at center) (1)
    Prepared at central kitchen operated by center(s) (2)
    Prepared by school food service (3)
    Pre-prepared by and purchased from independent food service company (4)
    Other (write in) (5)

16. How long has your organization been open for operation?
    Less than 6 months (1)
    6 months up to 1 year (2)
    1 year up to 3 years (3)
    3 years up to 5 years (4)
    5 years up to 10 years (5)
    10 or more years (6)

17. How long has your center participated in CACFP?
    Less than 6 months (1)
    6 months up to 1 year (2)
    1 year up to 3 years (3)
    3 years up to 5 years (4)
    5 years up to 10 years (5)
    10 or more years (6)

18. *Has your center ever participated in CACFP through a CACFP sponsoring organization? (e.g. not through a direct contract with the State)
    Yes (1)
    No (0)
    Unsure (2)
    *Question only asked of participants of Focus Group 1 – Independent child care centers that contract directly with the state to operate CACFP.

19. *Has your center ever participated in CACFP directly with the State (e.g. not through a CACFP sponsoring organization)?
    Yes (1)
    No (0)
    Unsure (2)
    *Question only asked of participants of Focus Group 2 – Independent child care centers that operate CACFP through a sponsoring organization.

20. Do you get support on CACFP from any of the following? (choose all that apply)
    CACFP Roundtable (1)
    National CACFP Sponsors Association (NCA) (2)
    National CACFP Forum (3)
    USDA Team Nutrition (4)
    Institute of Child Nutrition (5)

CA Department of Social Services (CDSS) or CA Department of Education (CDE) (6)
Other (write in) (7)
None of the above (8)

21. Please answer the following questions about yourself.

22. What is your job title? (choose all that apply)
Center owner (1)
Director or Site supervisor (2)
Executive director (3)
Teacher (4)
Other (write in) (5)

23. What is your sex?
Male (1)
Female (2)
Non-binary (3)
Prefer not to say (4)

24. Are you Hispanic or Latinx?
Yes (1)
No (0)

25. How would you describe yourself? (choose all that apply)
Asian/Pacific Islander (1)
Black or African American (2)
Native American or American Indian (3)
White (4)
Other (write in) (5)

26. What is the highest level of education you have completed?
Less than high school (1)
High school graduate (2)
Some college or Associate's degree (3)
Bachelor's degree (4)
Master's degree or higher (5)

27. What is your preferred language?
English (1)
Spanish (2)
Chinese (3)
Other (write in) (4)

A.2. Survey for Focus Group 3 – Sponsors of Independent Child Care Centers that Operate the CACFP

1. Thank you for agreeing to participate in the California Department of Social Services Child and Adult Care Food Program transition focus group. As a reminder, we provided you a letter of information over email detailing the goals of this research study. Here is a link to that letter.
Please complete this brief online survey before you participate in the online Zoom video conference focus group.

Please click the arrow, below, to begin the survey.

2. Please answer the following questions about your sponsor organization.

3. What is the zip code of your organization?

4. Which counties does your organization serve? (choose all that apply)
Alameda (1)
Alpine (2)
Amador (3)
Butte (4)
Calaveras (5)
Colusa (6)
Contra Costa (7)
Del Norte (8)
El Dorado (9)
Fresno (10)
Glenn (11)
Humboldt (12)
Imperial (13)
Inyo (14)
Kern (15)
Kings (16)
Lake (17)
Lassen (18)
Los Angeles (19)
Madera (20)
Marin (21)
Mariposa (22)
Mendocino (23)
Merced (24)
Modoc (25)
Mono (26)
Monterey (27)
Napa (28)
Nevada (29)
Orange (30)
Placer (31)
Plumas (32)
Riverside (33)
Sacramento (34)
San Benito (35)
San Bernardino (36)
San Diego (37)
San Francisco (38)
San Joaquin (39)
San Luis Obispo (40)
San Mateo (41)
Santa Barbara (42)
Santa Clara (43)

Santa Cruz (44)
Shasta (45)
Sierra (46)
Siskiyou (47)
Solano (48)
Sonoma (49)
Stanislaus (50)
Sutter (51)
Tehama (52)
Trinity (53)
Tulare (54)
Tuolumne (55)
Ventura (56)
Yolo (57)
Yuba (58)

5. What is the total number of staff at your organization (counting yourself)?
   Within your entire organization: (4)
   Within your CACFP department (5)

6. What languages does your organization support for your center directors?
   English (1)
   Spanish (2)
   Chinese (3)
   Other (4)
   Yes (1)
   No (0)
   Don't Know (2)

7. How long has your organization been open for operation?
   Less than 6 months (1)
   6 months up to 1 year (2)
   1 year up to 3 years (3)
   3 years up to 5 years (4)
   5 years up to 10 years (5)
   10 or more years (6)

8. Please select the appropriate response for your organization.
   Private, for profit (1)
   Private, non-profit (2)
   Government (3)
   Other (write in) (4)

9. Do you operate any other child nutrition programs? (choose all that apply)
   At-risk / afterschool program (1)
   Summer Food Service Program (2)
   National School Lunch Program (3)
   School Breakfast Program (4)
   Other (write in) (5)

10. Do you get support on CACFP from any of the following? (choose all that apply)
    CACFP Roundtable (1)
    National CACFP Sponsors Association (NCA) (2)
    National CACFP Forum (3)
    USDA Team Nutrition (4)
    Institute of Child Nutrition (5)
    CA Department of Social Services (CDSS) or CA Department of Education (CDE) (6)
    Other (write in) (7)
    None of the above (8)

11. Please answer the following questions about yourself.

12. What is your job title? (choose all that apply)
    Executive Director (1)
    Monitor / Field Representative (2)
    Office Support (3)
    Supervisor (4)
    Other (write in) (5)

13. What is your sex?
    Male (1)
    Female (2)
    Non-binary (3)
    Prefer not to say (4)

14. Are you Hispanic or Latinx?
    Yes (1)
    No (0)

15. How would you describe yourself? (choose all that apply)
    Asian/Pacific Islander (1)
    Black or African American (2)
    Native American or American Indian (3)
    White (4)
    Other (write in) (5)

16. What is the highest level of education you have completed?
    Less than high school (1)
    High school graduate (2)
    Some college or Associate's degree (3)
    Bachelor's degree (4)
    Master's degree or higher (5)

17. What is your preferred language?
    English (1)
    Spanish (2)
    Chinese (3)
    Other (write in) (4)
