# Supplementary material for: Child and Adult Care Food Program Participation Benefits, Barriers and Facilitators for Independent Child Care Centers in California
Source: Nutrients. 2022 Oct 22;14(21):4449. doi: 10.3390/nu14214449 (PMC9654096; doi:10.3390/nu14214449)
Supplement: Supplementary file 1 [file nutrients-14-04449-s001.zip › File S2.pdf]

Focus Group/Interview Questions

B.1. Questions for Focus Group 1 – Independent Child Care Centers that Contract Directly with the State to Operate the CACFP

1. Why are you participating in CACFP?
    a. Why do you not go through a sponsoring agency?

2. What are the greatest challenges to participating in CACFP?

3. What are the greatest technological barriers to participating in CACFP?

4. Has your center ever discontinued participating in CACFP?

5. What sort of state technical assistance would enable you to more easily participate in CACFP?

B.2. Questions for Focus Group 2 – Independent Child Care Centers that Operate the CACFP Through a Sponsoring Organization

1. Why are you participating in CACFP?

2. What are the greatest challenges to participating in CACFP?

3. What are the greatest technological barriers to participating in CACFP?

4. Has your center ever discontinued participating in CACFP?

5. What sort of sponsor technical assistance would enable you to more easily participate in CACFP?

B.3 Questions for Focus Group 3 – Sponsors of Independent Child Care Centers that Operate the CACFP

1. What barriers do centers experience participating in CACFP?

2. What are the greatest technological barriers to participating in CACFP?

3. Why do some centers not participate in the CACFP program?
    a. Why have centers that were previously on CACFP dropped off the program?

4. What sort of state technical assistance would enable you to more effectively serve center providers, children and families?

5. What does a supportive state agency look like?

6. How can the state support the centers that you sponsor to participate in CACFP?
